# EVALUATION OF A PROVIDER-DRIVEN STRATEGY TO ENSURE AGE-FRIENDLY CARE

**DOI:** 10.1093/geroni/igad104.2704

**Published:** 2023-12-21

**Authors:** Heba AlDossary, Anna Bender, Anne Pohnert, Mary Dolansky

**Affiliations:** Frances Payne Bolton, Case Western Reserve University, Cleveland, Ohio, United States; University of Washingotn, Seattle, Washington, United States; CVS Health, Bellingham, Massachusetts, United States; Case Western Reserve University, Cleveland, Ohio, United States

## Abstract

Implementation of evidence-based care requires innovation. The Age-Friendly Health Systems Questionnaire (AFHS-Q) is an example of a front-line innovation that increases efficiency of implementation by having the patient complete eight 4Ms questions while waiting for their visit. We evaluated the usability, acceptability, feasibility, and effectiveness of the AFHS-Q by surveying nurse practitioners (NPs) across a retail clinic system and found that the innovative practice-based tool was effective and feasible. The AFHS-Q kit was sent to over 1,100 retail clinic sites and an online survey was sent four weeks later to the 3,300 NPs at these sites to measure their experiences. NPs (N = 100) completed the survey and reported overall positive feedback (scale range 1-5) about the AFHS-Q relating to comfort of use (M = 4.18, SD = .99), usefulness (M = 3.97, SD = 1.10), acceptability (M = 4.12, SD = .83), and effort for use (M = 2.75, SD = 1.17). NP’s confidence in completing the 4Ms (before AFHS-Q: M = 3.92, SD = 1.90; after AFHS-Q: M = 4.18, SD = .97) and documenting the 4Ms (before AFHS-Q: M = 3.84, SD = .99; after AFHS-Q: M = 4.17, SD = .91) was significantly higher following AFHS-Q use (completion: t = 3.58, p < .001; documentation: t = 4.33, p < .001). NPs reported a significant reduction in time for visits after using the AFHS-Q. Participants also provided open ended feedback indicating the AFHS-Q was overall easy to use as well as highlighting areas for other improvement.

